# Harnessing High Density Lipoproteins to Block Transforming Growth Factor Beta and to Inhibit the Growth of Liver Tumor Metastases

**DOI:** 10.1371/journal.pone.0096799

**Published:** 2014-05-05

**Authors:** José Medina-Echeverz, Jessica Fioravanti, Nancy Díaz-Valdés, Kathrin Frank, Fernando Aranda, Celia Gomar, Nuria Ardaiz, Javier Dotor, Viktor Umansky, Jesús Prieto, Pedro Berraondo

**Affiliations:** 1 Division of Hepatology and Gene Therapy, Center for Applied Medical Research, University of Navarra, Pamplona, Spain; 2 Skin Cancer Unit, German Cancer Research Center, Heidelberg and Department of Dermatology, Venereology and Allergology, University Medical Center Mannheim, Ruprecht-Karl University of Heidelberg, Mannheim, Heidelberg, Germany; 3 DIGNA Biotech, Madrid, Spain; 4 Liver Unit, University of Navarra Clinic, Networked Biomedical Research Center of Hepatic and Digestive Diseases, Pamplona, Spain; University of Texas Health Science Center, United States of America

## Abstract

Transforming growth factor β (TGF-β) is a powerful promoter of cancer progression and a key target for antitumor therapy. As cancer cells exhibit active cholesterol metabolism, high density lipoproteins (HDLs) appear as an attractive delivery system for anticancer TGFβ-inhibitory molecules. We constructed a plasmid encoding a potent TGF-β-blocking peptide (P144) linked to apolipoprotein A-I (ApoA-I) through a flexible linker (pApoLinkerP144). The ApoLinkerP144 sequence was then incorporated into a hepatotropic adeno-associated vector (AAVApoLinkerP144). The aim was to induce hepatocytes to produce HDLs containing a modified ApoA-I capable of blocking TGF-β. We observed that transduction of the murine liver with pApoLinkerP144 led to the appearance of a fraction of circulating HDL containing the fusion protein. These HDLs were able to attenuate TGF-β signaling in the liver and to enhance IL-12 -mediated IFN-γ production. Treatment of liver metastasis of MC38 colorectal cancer with AAVApoLinkerP144 resulted in a significant reduction of tumor growth and enhanced expression of IFN-γ and GM-CSF in cancerous tissue. ApoLinkerP144 also delayed MC38 liver metastasis in *Rag2^−/−^IL2rγ^−/−^* immunodeficient mice. This effect was associated with downregulation of TGF-β target genes essential for metastatic niche conditioning. Finally, in a subset of *ret* transgenic mice, a model of aggressive spontaneous metastatic melanoma, AAVApoLinkerP144 delayed tumor growth in association with increased CD8^+^ T cell numbers in regional lymph nodes. In conclusion, modification of HDLs to transport TGF-β-blocking molecules is a novel and promising approach to inhibit the growth of liver metastases by immunological and non-immunological mechanisms.

## Introduction

Transforming growth factor β (TGF-β) has long been recognized as a key promoter of tumor growth and a critical initiator of the metastatic process [1]. Most established tumors develop mutations in the TGF-β-signaling pathway. These mutations confer the tumor cells the possibility of bypassing TGF-β–mediated growth inhibition while making use of the ability of this cytokine to enhance tumor growth, invasion, and metastasis [2]. TGF-β is a potent inducer of epithelial-mesenchymal transition (EMT) [3], a process by which tumor cells lose epithelial characteristics and acquire enhanced migratory and invasive capabilities. Moreover, TGF-β directly promotes the expression of key angiogenic factors such as VEGF [4] and induces metalloproteases like MMP9 [5], facilitating tumor vascularization and matrix remodeling which are critical events involved in conditioning the metastatic niche [6]. TGF-β also stimulates tumor invasion and metastasis by inhibiting anti-tumor immune surveillance and promoting local immune suppression. Along this line, TGF-β directly inhibits NK and T cell proliferation and suppresses CD8^+^ T cell cytotoxicity by transcriptional repression of genes encoding perforin, granzymes and cytotoxins [7]. In addition, TGF-β induces FoxP3 thereby generating inducible regulatory T lymphocytes and exerts additional immunosuppressive functions by acting on myeloid-derived suppressor cells, tumor-associated neutrophils and tumor-associated macrophages [1], [7]. Thus, TGF-β is a polyvalent cytokine that favors tumor growth and spread by a diversity of mechanisms. Accordingly, antitumor strategies directed to effectively block TGF-β have long been sought. However, attempts to antagonize TGF-β in malignant processes were not followed by clear clinical benefit and clinical development of two of the three products which reached phase II were discontinued [8].

We have previously reported that a lipophilic 14-mer peptide derived for type III TGF-β receptor, termed P144, is a potent TGF-β inhibitor that displays therapeutic effects in models of liver fibrosis [9], scleroderma [10] and myocardial fibrosis [11]. In the present work, we tested the effect of P144 in animal models of metastatic liver cancer. To ensure sustained serum levels of the bioactive molecule, we used a liver-directed gene therapy approach employing adeno-associated viral vectors (AAV) which enable long-term expression of the transgene. As tumors exhibit an active cholesterol metabolism and express high levels of the ApoA-I receptor, SRB1 [12], [13], we constructed a transgene formed by p144 fused to ApoA-I through a flexible linker (ApoLinkerP144). Consonant with the fact that ApoA-I is the main protein of HDLs, here we show that transduction of the liver with a vector encoding ApoLinkerP144 leads to the secretion of HDLs with TGF-β blocking properties and that this strategy is an efficient therapy to inhibit the growth of liver tumor metastases.

## Materials and Methods

### Cell lines

MT\Ret melanoma was described in [14]. MC38 is a colon adenocarcinoma cell line of C57BL/6 origin whose identity has been verified by Idexx Radil (Columbia, MO, USA. Case 6592–2012) and was provided to us by Dr. Karl E. Hellström (Seattle, WA) [15]. Mv-1-Lu lung carcinoma (ATCC CCL-64) and mouse colon carcinoma cell lines CT26 (ATCC CRL-2638) were obtained from American Type Culture Collection (Manassas, VA, USA). Cells were maintained in RPMI 1640 with or without GlutaMAX respectively and supplemented with 10% heat-inactivated fetal calf serum, 100 units/ml penicillin, 100 μg/ml streptomycin and 5×10^−5^ mol/l 2-mercaptoethanol (RPMI complete medium).

### Ethics Statement

This study was carried out in strict accordance with the institutional guidelines for animal care of the University of Navarra and German Cancer Research Center (DKFZ). The protocol was approved by the Committee on the Ethics of Animal Experiments of the University of Navarra and by the Committee on the Ethics of Animal Experiments of the German Cancer Research Center (DKFZ) (experiment with ret mice). All surgery was performed under isofluorane anesthesia, and all efforts were made to minimize suffering. Mice were sacrificed by CO_2_ inhalation.

### Mice

Five to seven-week-old female BALB/c and C57BL/6 mice (Harlan Laboratories, Barcelona, Spain), *Rag2^−/−^IL2rγ^−/−^* and *ApoA-I^−/−^* mice were kept in the University of Navarra animal facilities and cared for according to the institutional guidelines for animal care. C57BL/6 mice expressing human *ret* oncogene in melanocytes were provided by I. Nakashima (Chubu University, Aichi, Japan) and experiments were performed in accordance with German government and DKFZ institutional guidelines and regulations.

### Animal manipulation

Hydrodynamic administration of plasmids was performed with 20 µg purified plasmids prepared with EndoFree Maxi Kit (Qiagen, Valencia, CA). IL-12 was expressed under the control of a liver-specific Dox-inducible system. Doxycycline (Sigma, Madrid, Spain) was given to IL-12 treated animals at a concentration of 2 mg/ml in drinking water containing 5% sucrose (Panreac, Barcelona, Spain). Blood samples were obtained by retro-orbital bleeding and serum was obtained and stored at –20°C until assayed.

For the model of colorectal metastasis in the liver, 5×10^5^ MC38 cells were inoculated in the spleen after laparotomy. Once mice recovered from anesthesia, AAV vectors were inoculated in 200 µl saline by intraperitoneal injection. *Rag2^−/−^IL2rγ^−/−^* mice were sacrificed at day 10 and C57BL/6 at day 15. Tumor area in images of the livers was measured quantifying the pixels over a threshold color using Matlab software (Matworks, Natick, MA, USA). Samples from healthy liver and tumors were stored at −80°C until assayed.

5 week-old *Ret* transgenic tumor-bearing mice received either saline or AAVApoLinkerP144 by intraperitoneal injection. Both groups were monitored daily for tumor progression to assess survival. In a set of experiments, remaining mice were sacrificed at day 50 upon treatment. Responder and non-responder classification of AAVApoLinkerP144–treated mice was done *post-hoc* regarding tumor weight. Fresh tumor and lymph node samples were mechanically dissociated in ice-cold PBS and filtered through cell strainers (BD Biosciences, San Jose, CA, USA). Samples were depleted of erythrocytes by ammonium chloride lysis, washed twice and stored on ice.

### CD3 and pAKT immunostaining

For CD3 quantification, liver samples containing tumor nodules were embedded in Tissue-Tek OCT (Sakura, Zoeterwoude, The Netherlands). Samples were air dried, and fixed in 4% formaldehyde for 5 min. Endogenous peroxidase was quenched with 3% H_2_O_2_ in methanol for 10 min. Tissue sections were incubated o/n at 4°C with anti-human CD3 (clone SP7, Lab Vision Corporation, Fremont, CA) diluted 1∶100 in TBS. Then, sections were incubated with anti-rabbit EnVision HRP-polymer (Dako, Carpinteria, CA) and revealed using DAB+ (Dako). Sections were lightly counterstained with Harris hematoxilin. For pAKT evaluation, formalin-fixed paraffin-embedded liver sections (3 µm thick) were dewaxed and hydrated. Antigen retrieval was performed for 30 min at 95°C in 0,01 M Tris-1 mM EDTA (pH = 9) in a Pascal pressure chamber (S2800, Dako). Endogenous peroxidase was blocked with 3% H_2_O_2_ in deionized water. Sections were incubated overnight at 4°C with anti-phosphoAKT (1∶50, Cell Signaling, 4060). After rinsing in TBS-T, the sections were incubated with goat anti-rabbit EnVision (Dako; Glostrup, Denmark) for 30 min and peroxidase activity was revealed using DAB+ (Dako). Finally, sections were lightly counterstained with Harris hematoxylin, dehydrated, and coverslipped.

### Antibodies and flow cytometry

Rabbit anti phospho-Smad2 (Millipore, Billerica, MA, USA), rabbit anti Smad2/3 (Cell Signaling Technology, Danvers, MA, USA), rabbit anti phospho-Smad3 (Cell Signaling Technology), goat anti phospho-Smad1/5/8 (Santa Cruz Biotechnology, Santa Cruz, CA, USA), rabbit anti Smad1/5/8 (Santa Cruz Biotechnology), rabbit anti-mouse phospho-Stat1 (Cell Signaling Technology), rabbit anti-mouse ApoA-I (Santa Cruz Biotechnology), rabbit anti-mouse Actin (Sigma, Steinheim, Germany) and HRP conjugate donkey anti-rabbit IgG (Southern Biotech, Birmingham, AL, USA) were used for western blot analysis. Rabbit anti SRB1 monoclonal antibody (Novus Biologicals, CO, USA) and FITC conjugated anti-rabbit IgG (Sigma, Steinheim, Germany) were used for flow cytometry analysis. Fc block and fluorochrome conjugated antibodies against mouse CD45, CD3, CD8, MHC-II, CD11c and TNF-alpha were purchased from BD Biosciences. Intracellular staining for TNF-alpha was performed following manufacturer's instructions. For flow cytometry experiments acquisition was done using either FACSCalibur or FACSCanto II (BD Biosciences). FlowJo Software (TreeStar) was used to analyze cellular events.

### Plasmids

pIL-12 and pApo have been previously described [16], [17]. To obtain the plasmid pApoLinkerP144, we used the plasmid pCMV-mApoA-IAscI [16] digested with AscI and NotI. FwLinkerP144 and RvLinkerP144 primers were then hybridized, leaving cohesive ends that match with AscI excision site in 5′ and NotI in 3′. This primer introduced a flexible linker sequence derived from pyruvate ferredoxin oxidoreductase between ApoA-I and P144. The plasmid pSpP144, with the signal peptide of ApoA-I followed by the coding sequence of P144, was obtained by PCR with primers FwATGmApoA-I and RvSPmApoAIP144 and as a template, pCMV-mApoA-IAscI. Primers are listed in Table S1.

### Adeno-associated virus (AAV) production

All AAV plasmids contain an expression cassette flanked by two inverted terminal repeats from the AAV2. The expression cassette contains the ubiquitous human 1-α elongation factor (EF1-α) promoter, the gene encoding for mouse ApoA-I (*apoa1*, GeneID:11806) or the cDNA encoding for ApoLinkerP144 and the bovine growth hormone polyadenylation sequence [bGH poly(A)] (bases 2,326–2,533: GenBank accession no. M57764). AAV2/8 vectors were produced by calcium phosphate–mediated transfection of HEK-293T cells. The resulting viral vectors (AAVApo or AAVApoLinkerP144) were harvested and purified by iodixanol-gradient centrifugation followed by filtration and further concentration against phosphate- buffered saline–5% sacarose. Viral titers in terms of genome copies per milliliter (gc/ml) were determined by quantitative polymerase chain reaction.

### qRT-PCR

Total RNA either from mice livers or from liver tumor nodules was isolated using TRI reagent (Sigma, Dorset, UK). RNA was treated with DNase I and retrotranscribed to cDNA with MMLV RT in the presence of RNase OUT (all reagents from Invitrogen, Carlsbad, CA, USA) according to manufacturer's instructions. Primers for quantitative real time RT-PCR are listed in Table S1 in File S1. H3F3 histone was used to standardize gene expression. The mRNA values were represented by the formula 2Δ^Ct^, where ΔCt indicates the difference in the threshold cycle between H3F3 histone and the target genes (all reagents from BioRad, Hercules, CA, USA).

### Reagents

Peptide SPSYVYHQF (hereafter AH1) is a cytotoxic T cell determinant expressed by CT26 cells and presented by H-2L^d^ MHC class I molecules was purchased from Proimmune Ltd. (Oxford, United Kingdom). IL-12p70, interferon gamma (both BD Biosciences) and mouse ApoA-I (Cusabio Biotech Ltd.) serum levels were quantified by ELISA kits according to manufacturer's instructions. Peptide P144 (TSLDASIIWAMMQN) was obtained from Digna Biotech (Madrid, Spain). Recombinant ApoLinkerP144 was produced and purified by Genscript (Piscataway, NJ, USA).

### HDL isolation by sodium bromide gradient differential ultracentrifugation

24 hours after hydrodynamic delivery of pApo or pApoLinkerP144 plasmids, HDL isolation was performed from pool plasma samples as described by Fioravanti *et al.* [16].

### In vitro assay of the TGF-β blocking activity of HDL using Mv-1-Lu cells

Mv-1-Lu cells (5000 cells/well) were cultured in a 96-well flat-bottomed plate (Costar Corporation CA, USA) at 37°C, 5% CO_2_ overnight in complete medium (RPMI1640 containing L-glutamine and supplemented with 5% fetal calf serum and antibiotics). HDLs (10 µg/ml) were tested by triplicate in the presence of 200 pg/ml TGFβ1 (R&D, Minneapolis, MN). Cell proliferation after 72 h was determined using ViaLight Plus Kit (Lonza, Rockland, ME, USA). As positive and negative controls, we used Mv-1-Lu cells grown in absence or presence of human TGF-β, respectively. Percentage of inhibition of TGF-β was calculated normalizing data with the positive and negative controls.

### Statistical analysis

Prism software (GraphPad Software, Inc.) was employed to perform statistical analysis. Log-rank test was used to determine the significance of differences in survival curves and percentage of tumor-free mice. Differences in other parameters among groups were analyzed by Student's T test or with one-way ANOVA followed either by Dunnett's or by Bonferroni's posttest. *P* values <0.05 were considered to be statistically significant.

## Results

### Liver transduction with a plasmid encoding the fusion protein ApoA-I/P144 results in the secretion of HDLs with TGF-β blocking properties

TGF-β is known to be a key driver of tumor metastases [2]. In this work, we sought to use P144, a potent anti-TGF-β peptide, to inhibit the growth of metastatic liver cancer. Since synthetic peptides have short half-life in the circulation, we tried to solve this problem by transducing the liver with genetic constructs encoding P144. Two plasmids were used: one containing P144 bound to a secretory signal peptide (pSpP144) and another with P144 linked to ApoA-I through a flexible linker (pApoLinkerP144). In addition, we employed a plasmid encoding ApoA-I (pApo) to serve as a control. We decided to fuse P144 to ApoA-I as this molecule is the main component of high density lipoproteins (HDL) and we have previously shown that when hepatocytes are compelled to express a cytokine linked to ApoA-I, the fusion protein circulates in plasma incorporated into HDLs [16]. We reasoned that conveyance of P144 by HDLs might favour its antitumor activity as cancer cells exhibit an active cholesterol metabolism and express high levels of the ApoA-I receptor, SRB1 [12], [13].

In preliminary experiments, we tested the TGF-β inhibitory activity of the different constructs using an *in vivo* assay based on the ability of TGF-β to inhibit interleukin 12 (IL-12)-mediated interferon-γ (IFN-γ) production [18]. Thus, we transduced the liver of BALB/c mice by hydrodynamic co-injection of a plasmid encoding IL-12 (pIL-12) together with pApo, pSpP144, or pApoLinkerP144. We found that only pApoLinkerP144 was able to increase intrahepatic IFN-γ expression following liver transduction with pIL-12 (Figure S1A in File S1). We also analyzed the adjuvant activity of pApo, pSpP144 and pApoLinkerP144 in prophylactic vaccination of BALB/c mice with the AH-1 peptide derived from CT26 colon cancer, a setting where TGF-β blockade has been shown to enhance antitumor immune responses [19]. We found that after subcutaneous implantation of CT26 cells the tumor developed in all vaccinated mice treated with pApo or pSpP144 while those which received pApoLinkerP144 remained tumor-free (Figure S1B in File S1). Thus, our data showed that only pApoLinkerP144 exhibited immunostimulatory properties likely derived from its anti-TGF-β activity. Thus, we continued the characterization of ApoLinkerP144 using ApoA-I as control.

**Figure 1:**
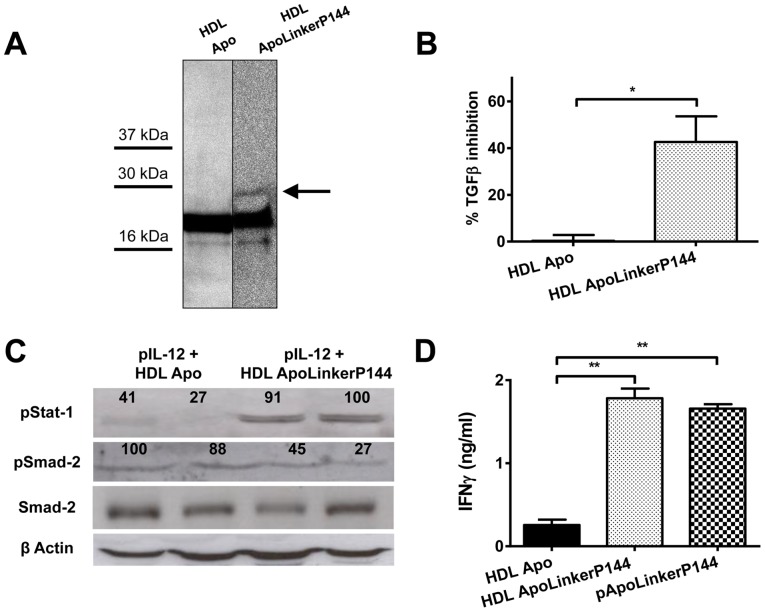
Blockade of TGF-β *in vivo* by ApoLinkerP144 delivered either by gene therapy or bound to HDL. (A) Western blot against ApoA-I with purified HDL fractions of plasma obtained from animals which received 24 h before either pApo or pApoLinkerP144 by hydrodynamic injection. (B) Inhibition of TGF-β by HDL from mice injected with Apo plasmid (HDL Apo) or HDL containing ApoLinkerP144 (HDL ApoLinkerP144) measured using Mv-1-Lu cells cultured in the presence of 200 pg/ml of TGF-β and 10 µg/ml HDLs. Mean±SEM *, *P*<0.05 (C) Strengthened pStat-1 expression and diminished pSmad-2 expression in livers from mice which received IL-12 plasmid and HDL Apo or HDL ApoLinkerP144. Numbers indicate the relative intensity of the bands. (D) IFN-γ production 4 days after IL-12 plasmid hydrodynamic injection combined with i.p injection of HDL Apo or HDL ApoLinkerP144 or a hydrodynamic injection of pApoLinkerP144 in C57BL/6 mice (*n* = 4 mice/group). Mean±SEM **, *P*<0.01.

To see whether ApoLinkerP144 circulated incorporated into HDLs, mice were treated by hydrodynamic injection with pApo or pApoLinkerP144 and 24 hours later, the HDL fraction of plasma was isolated and subjected to Western blot using an anti-ApoA-I antibody. We observed that mice that received pApoLinkerP144 showed an additional band of higher molecular weight than ApoA-I likely corresponding to the fusion protein (Figure 1A). Then, we analyzed whether HDLs from pApoLinkerP144-treated mice were able to block TGF-β activity *in vitro* using a bioassay based on the ability of TGF-β to inhibit the proliferation of the Mv-1-Lu cells. As shown in Figure 1B, HDLs from pApoLinkerP144-treated mice were able to partially restore the proliferation of Mv-1-Lu cells incubated in the presence of TGF-β. Moreover, we analyzed the inhibitory activity of a recombinant ApoLinkerP144 on the TGF-β signaling in MC38 cells. We observed a reduction in both Smad2 and 3 phosphorylation (Figure S2 in File S1). To analyze whether HDLs containing ApoLinkerP144 could block TGF-β *in vivo*, C57BL/6 mice were subjected to hydrodynamic injection of pIL-12 and injected intraperitoneally at the same time with HDLs isolated from mice that had received pApo or pApoLinkerP144. We found that co-administration of pIL-12 plus ApoLinkerP144-containing HDLs was able to attenuate Smad2 phosphorylation while enhancing Stat1 activation and therefore IFN-γ production in serum (Figure 1C, D). These data show that HDL containing ApoLinkerP144 attenuates TGF-β signaling and stimulates IFN-γ production within the liver.

### An adeno-associated viral vector encoding ApoLinkerP144 enables sustained *in vivo* transgene expression and displays anti-tumor activity in a model of liver metastasis

**Figure 2:**
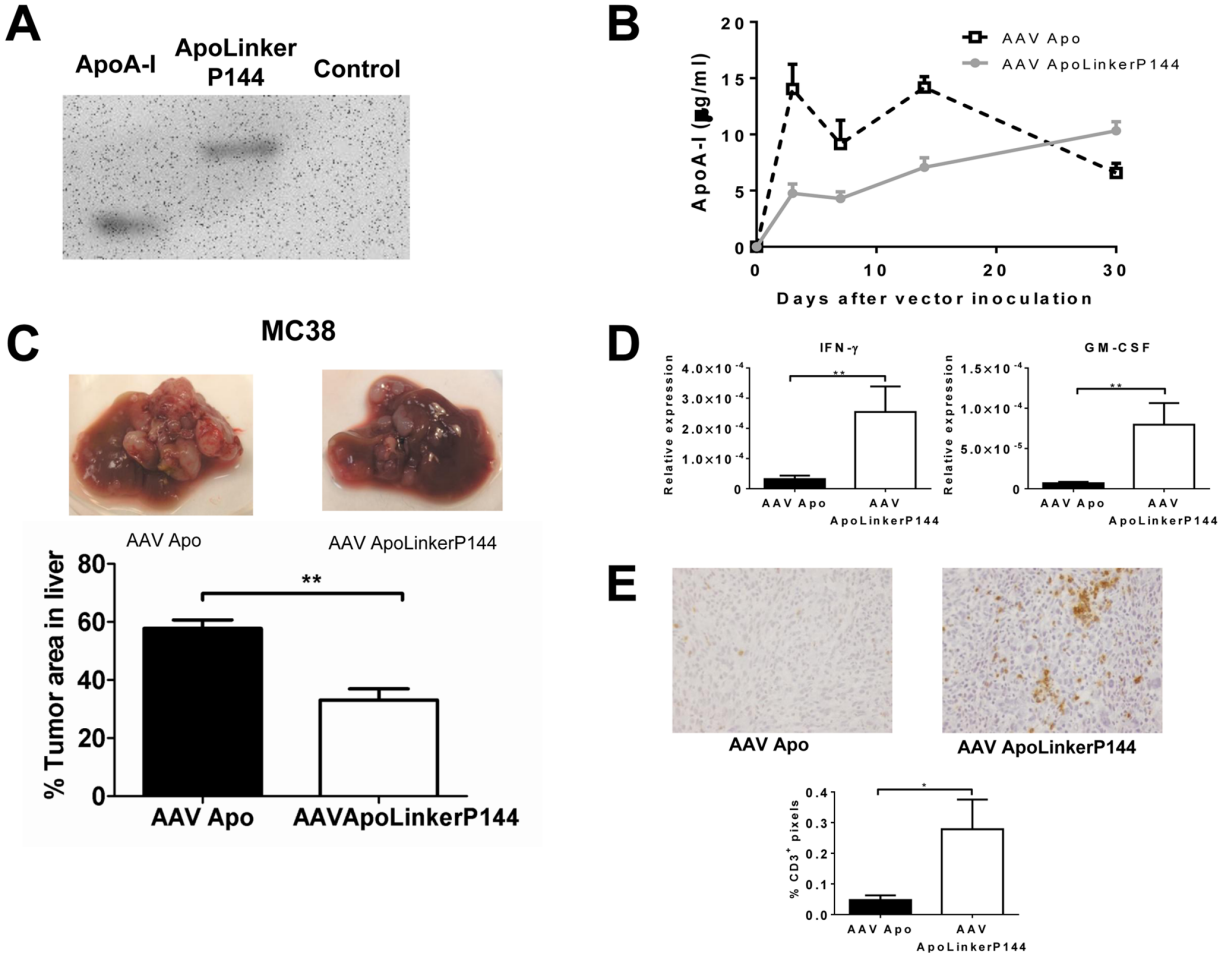
Sustained expression of ApoLinkerP144 in the liver using an AAV vector significantly diminishes the occurrence of primary liver metastasis. (A) ApoA-I Western blot analysis performed on supernatants of 293T cells producing the AAV vectors. (B) ApoA-I deficient mice received 4×10^11^ genome copies/mice i.v. (*n* = 6/group) and ApoA-I production in serum was quantified by ELISA. (C) 5×10^5^ MC38 colon carcinoma cells were intrasplenically injected. At the same time, 4×10^12^ AAVApo or AAVApoLinkerP144 vg/mice were i.p injected (*n* = 6/group). Mice were sacrificed at day 15 after tumor cell inoculation and tumor area in the liver was measured quantifying the pixels over a threshold color using Matlab software. Mean±SEM **, *P*<0.01. (D) IFNγ and GM-CSF expression in metastatic nodules in the liver was assessed by RT-PCR. Mean±SEM *, P<0.05. **, P<0.01 (E) Representative pictures of immunohistochemical staining of CD3 in the metastatic nodules in the liver (Magnification: 200×) and quantification of CD3^+^ pixels per field in ten fields per animal quantifying the pixels over a threshold color using Matlab software (Magnification: 100×). Mean±SEM *, P<0.05.

We then tested whether sustained expression of ApoLinkerP144 in hepatic tissue could exert antitumor effects in a model of metastatic colorectal cancer in the liver. To this aim, we selected a recombinant AAV vector to express ApoA-I or ApoLinkerP144 in the liver as these vectors allow long term transgene expression and have already been used in humans with good tolerance [20]. We employed AAV serotype 8 because among all serotypes this is the one with the highest liver transduction efficiency in mice [21]. Thus, we produced AAV vectors encoding ApoA-I (AAVApo) or ApoLinkerP144 (AAVApoLinkerP144) and the expression of these molecules was verified by Western blot analysis in supernatants of 293T-HEK cells transfected with the plasmids used to generate the respective vectors (Figure 2A). Next, AAVApo or AAVApoLinkerP144 at the dose of 4×10^12^ vg/mice were injected intravenously in ApoA-I deficient mice and serum ApoA-I was quantitated by ELISA at different time points. We observed that with either of the two vectors serum levels of the transgene were maintained at values above 4 µg/ml during at least one month (Figure 2B).

Then, we studied whether AAVApoLinkerP144 could affect the growth of metastatic lesions in the liver of C57BL/6 mice following intrasplenic injection of 5×10^5^ MC38 colon cancer cells. This tumor cell line was chosen as it is representative of SRB1 expressing tumors (Figure S3 in File S1). After administration of the neoplastic cells, the animals received AAVApoLinkerP144 (4×10^12^ vg/mice) or the same dose of AAVApo by intraperitoneal injection. We observed a significant reduction of MC38 metastatic lesions in mice treated with AAVApoLinkerP144 compared to those given AAVApo (Figure 2C). A similar antitumor effect was observed after administration of 200 µg P144 in carbonate buffer (pH 9.5) every day, indicating that AAVApoLinkerP144 was an efficient delivery vehicle of the anti-TGFβ peptide (Figure S4 in File S1).

**Figure 3:**
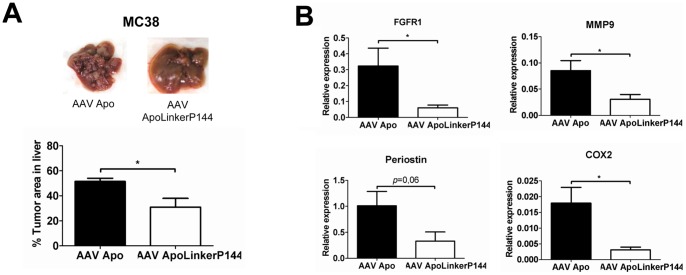
Hepatic tumor nodule incidence by ApoLinkerP144 is also diminished in immunodeficient mice by modulating TGF-β–dependent molecules. (A) 5×10^5^ MC38 colon carcinoma cells were intrasplenically injected and 4×10^12^ AAVApo or AAVApoLinkerP144 vg/mice were simultaneously i.p injected in *Rag2^−/−^IL2rγ^−/−^* mice (*n* = 6/group). Mice were sacrificed at day 10 after the tumor cell inoculation and the tumor area in the liver was measured quantifying the pixels over a threshold color using Matlab software. Mean±SEM *, *P*<0.05. (B) FGFR1, MMP9, periostin and COX-2 expression in tumor liver metastases from *Rag2^−/−^IL2rγ^−/−^* mice was assessed by RT-PCR. Mean±SEM *, *P*<0.05.

TGF-β plays a key role in cancer progression by suppressing antitumor immunity but also by inducing tumor cell dedifferentiation, EMT and metastatic behavior [22]. Thus, we explored whether the antitumor effect of AAVApoLinkerP144 was associated with modulation of immune and/or non-immune processes. We found that immunocompetent mice with hepatic metastasis of MC38 cancer which received AAVApoLinkerP144 showed enhanced expression of IFN-γ and GM-CSF in the metastatic nodules compared to those treated with AAVApo (Figure 2D). Indeed, immunohistochemical analysis showed an increase in the number of tumor infiltrating T cells in mice that received AAVApoLinkerP144 compared to those given AAVApo (Figure 2E). In addition, we also observed by immunohistochemical analysis a reduction in the phosphorylation of AKT in the tumor nodules of mice treated with AAVApoLinkerP144, reflecting that the fusion protein was able to block a non-canonical TGF-β signaling pathway *in vivo* (Figure S5 in File S1). Then, we analyzed whether AAVApoLinkerP144 could also inhibit liver metastasis in immunocompromised animals. To this aim, AAVApo or AAVApoLinkerP144 (4×10^12^ vg/mice) were administered to *Rag2^−/−^IL2rγ^−/−^* mice inoculated with MC38 cells into the spleen (Figure 3A). We found that mice treated with AAVApoLinkerP144 showed a significant reduction of tumor burden in the liver suggesting that non-immunological mechanisms contribute to the antitumor activity of this therapy. Indeed, different TGFβ-target genes including MMP9, COX-2 and FGFR1, which are critical for the conditioning of the metastatic niche [23], [24], [25], were significantly dowregulated in *Rag2^−/−^IL2rγ^−/−^* mice treated with AAVApoLinkerP144 compared to those that received AAVApo (Figure 3B). The mRNA levels of periostin, another TGFβ-inducible factor important for tumor progression [26], tended to be lower in the former group of animals being the difference at the limit of statistical significance (p = 0.06) (Figure 3B).

### AAVApoLinkerP144 exerts antitumor effects against spontaneous metastatic melanoma

**Figure 4:**
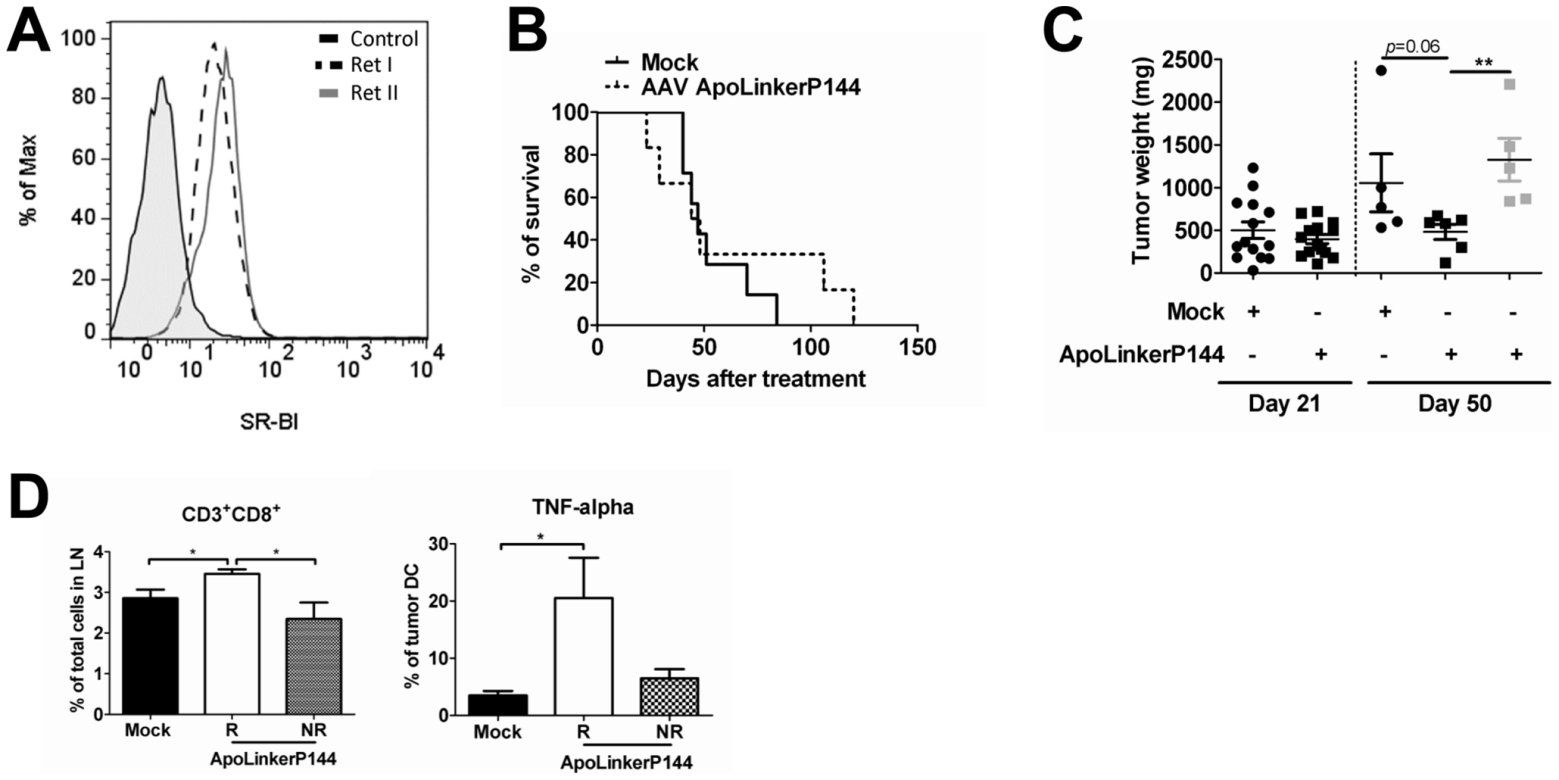
Sustained expression of ApoLinkerP144 delays tumor progression and increases intratumoral CD8 T cell infiltration in a mouse model of spontaneous melanoma. (A) Scavenger Receptor B class 1 (SRB1) expression on two different Ret cell lines (established from primary skin melanomas) analyzed by flow cytometry. (B) *Ret* transgenic tumor-bearing mice were injected i.p either with saline or 4×10^12^ gc/mouse of AAVApoLinkerP144 (*n* = 7/group). Results are displayed as Kaplan-Meier plot of mice survival. Cumulative data of two independent experiments are shown. (C) *Ret* transgenic mice were injected i.p either with saline or 4×10^12^ gc/mouse of AAVApoLinkerP144. Then, mice were sacrificed either at day 21 (*n* = 7/group) or 50 (saline, *n* = 5; treatment *n* = 11) after injection. Mice sacrificed at day 50 were classified as responder (R; grey bar, *n* = 6) or non-responder (NR; white bar, *n* = 5) regarding tumor weight; (D) Cells from primary skin tumors and metastatic lymph nodes were isolated at day 50 upon the treatment Frequency of CD8^+^ T lymphocytes in metastatic lymph nodes and of tumor-infiltrating dendritic cells producing TNF-α upon the treatment overnight with LPS measured by flow cytometry were presented as a percentage within the respective cell population; Mean±SEM *, *P*<0.05.

Having shown that ApoLinkerP144 inhibits liver metastases in a transplantable tumor model, we investigated the antitumor potential of ApoLinkerP144 in *ret* transgenic mice. These animals spontaneously develop skin malignant melanoma metastasizing to lymph nodes, lungs, brain, liver and the bone marrow [27], [28], [29]. This model was chosen since it is representative of a highly aggressive spontaneous metastatic tumor, and high SRB1 expression levels were found to be present in cell lines derived from this malignancy (Figure 4A). R*et* transgenic mice received a single dose of AAV ApoLinkerP144 or PBS (n = 7 per group) and the animals were followed for survival. In a different experiment, similar treatments were administered to the transgenic animals which were then sacrificed at day 21 (n = 7 per group) or 50 (PBS n = 7, 5 alive by day 50, treated n = 12, 11 alive). In the first experiment, all animals in the control group died by day 80 while 40% of those that received AAVApoLinkerP144 survived at day 100 although succumbed by day 120 (Figure 4B). In the second experiment, at day 50 after vector injection, 6 of the treated mice exhibited a liver tumor burden than tended to be reduced compared with mice injected with PBS (p = 0.06) while in the other 5 treated mice tumor progression was similar to controls (Figure 4C). The former group of 6 mice was designed as the responder group. Interestingly, responder mice presented a significant increase in the percentage of CD8^+^ T lymphocytes in the lymph nodes (Figure 4D). Moreover, the analysis of dendritic cells infiltrating the tumors showed that upon an overnight incubation with LPS (10 ng/ml), the expression of TNF-α was significantly higher in the responder group than in PBS -treated animals, while no differences were found between this last group and non-responders (Figure 4D). These data point to higher stimulation of adaptive immunity and stronger pro-inflammatory phenotype in antigen presenting cells of animals treated with AAVApoLinkerP144.

## Discussion

TGF-β is a pleiotropic cytokine that acts as a key driver of the metastatic process [2]. It mediates EMT and is essential for the preparation of the metastatic niche, for local conditioning of the extracellular matrix and for suppression of anti-tumor immunity [30]. It is therefore a relevant target for cancer therapy as its blockade would promote protective antitumor immune responses while simultaneously interfering with biological changes that are crucial for metastases progression [8]. P144 is a synthetic peptide that has been shown to attenuate liver fibrosis, scleroderma and miocardial esclerosis in different animal models (9–11). Importantly, local application of P144 as a cream has already been used in humans in a phase II clinical trial for skin fibrosis in systemic sclerosis [8]. Synthetic peptides, however, have very short half-life in circulation; a feature that limits their use as systemic therapy for conditions like malignancies. In order to maintain sustained plasma levels of the peptide, we transduced the liver with different constructs encoding P144. When using the peptide sequence bound to a secretory signal peptide, this construct did not markedly increase the IFN-γ induced by IL-12 and did not increased the immune response against a peptide-based vaccine. The lack of activity could be due to a defective secretion of the peptide or inadequate interaction of the secreted molecule with its target. In contrast, the fusion of P144 to ApoA-I through a flexible linker (ApoALinkerP144) resulted in the secretion to plasma of a fraction of HDLs with TGF-β blocking activity. In a previous report, we showed that the transduction of hepatocytes with a cytokine like IFN-α linked to ApoA-I caused the release to serum of the fusion protein incorporated into HDLs. In the present paper, we show that similarly happens when linking a short peptide such as P144 to ApoA-I. It should be noted that P144 is highly lipophilic and it seems possible that its bioavailability *in vivo* might be favored by becoming an integral part of lipoproteins.

As mentioned above, the idea of conveying the TGF-β inhibitor incorporated into HDLs is very attractive as cholesterol metabolism is essential for sustained cell growth and it is very active in tumors [12]. A great diversity of tumors, including colorectal cancer, express high levels of the ApoA-I receptor SRB1 which mediates the uptake of cholesteryl esters and other lipids from HDLs [13], [31], [32]. Indeed, the avidity of tumor cells for HDLs was soon appreciated and HDL and ApoA-I decorated particles have been used as drug carriers to deliver lipophilic drugs to tumors [13], [33].

Remarkably, administration of AAVApoLinkerP144 to immunocompetent mice exhibiting liver metastases induced a significant reduction of tumor burden in association with increased numbers of infiltrating T cells and upregulation of pro-inflammatory and immunostimulatory molecules such as GM-CSF and IFN-γ. The inhibition of liver metastasis was also observed in spontaneous tumors that overexpress SRB1 like the *ret* transgenic mouse model of aggressive spontaneous melanoma, which is very difficult to treat.

Importantly AAVApoLinkerP144 also reduced metastatic nodules in the liver of *Rag2^−/−^IL2rγ^−/−^* mice indicating that the antineoplastic effects of this vector are not only due to activation of antitumor immunity but also to interference with non-immune pro-tumorigenic activities of TGF-β. Indeed, in the metastatic lesions of these animals, we observed reduced expression of MMP9, COX-2, periostin and FGFR1. All these molecules are TGF-β targets and essential factors for tumor progression [23], [24], [25]. Therefore, AAVApoLinkerP144 appears to counteract metastatic tumor growth by way of both immune and no-immune mechanisms. These multifaceted actions have previously been described with other TGF-β inhibitors [34], [35].

In conclusion, AAVApoLinkerP144 allows the secretion by liver cells of HDLs with the ability to block TGF-β, representing a novel approach to combat liver metastasis. This treatment might prove to be synergistic with other targeted therapies or immunotherapies.

## Supporting Information

File S1 Contains the following files: Figure S1. *In vivo* assays to select the best anti-TGF-β inhibitor. (A) A plasmid encoding IL-12 (pIL-12) was administered to C57BL/6 mice by hydrodynamic injection together with pApo, pSpP144, or pApoLinkerP144. Four days later, IFN-γ serum levels were quantified by ELISA. **p<0.01. (B) BALB/c mice were immunized with AH-1 peptide emulsified in incomplete Freund's adjuvant. At day 7 after immunization, mice received plasmids pApoA-I, pSpP144 or pApoLinkerP144 via hydrodynamic injection. Seven days later, mice were injected s.c. with 2.5×10^5^ CT26 tumor cells. Results are displayed as Kaplan-Meier plot of tumor occurrence. Treatment groups were compared using the log-rank test. Data are representative of one of two independent experiments (n = 6 mice/group). *p<0.05.**p<0.01. Figure S2. Inhibitory effect of ApoLinkerpP144 on TGF-β signaling. 5×10^5^ MC38 cells were plated in a 6-well plates. After an overnight incubation, cells were left untreated or treated with 100 pM TGF-β with or without 100 µg/ml rApoLinkerP144. 60 minutes later, cells were harvested and Western blot analysis was performed to determine the phosphorylation status of Smad2, Smad3 and Smad1/5/8. Figure S3. Scavenger receptor class B type I (SRB1) expression on MC38 tumor cell line. 3×10^5^ tumor cells were cultured in 6-well plates for 48 hours. Then, cells were harvested and stained to show SRB1 surface expression by flow cytometry analysis. Expression was compared with isotype control. Data are representative of two independent experiments. Figure S4. Antitumor efficacy of P144. 5×10^5^ MC38 colon carcinoma cells were intrasplenically injected. The same day, we started a daily treatment with vehicle (carbonate buffer pH 9.5) or 200 µg P144 in carbonate buffer (pH 9,5) for 15 days (n = 6/group). Mice were sacrificed at day 15 after tumor cell inoculation and tumor area in the liver was measured quantifying the pixels over a threshold color using Matlab software. Mean±SEM *, P<0.05. Figure S5. Immunohistochemical staining of pAKT in the metastatic nodules in the liver. 5×10^5^ MC38 colon carcinoma cells were intrasplenically injected. At the same time, 4×10^12^ AAVApo or AAVApoLinkerP144 vg/mice were i.p injected (n = 6/group). Mice were sacrificed at day 15 after tumor cell inoculation and liver were fixed with formalin and embedded in paraffin. Immunhistochemical staining of pAKT was performed. A representative picture of pAKT in the metastatic nodules in the liver is shown (Magnification: 100×). Table S1. Primers designed to clone the different peptide fusions and the primers used for quantitative PCR analysis.
(DOC)
