# Supplementary material for: Harnessing High Density Lipoproteins to Block Transforming Growth Factor Beta and to Inhibit the Growth of Liver Tumor Metastases
Source: PLoS One. 2014 May 5;9(5):e96799. doi: 10.1371/journal.pone.0096799 (PMC4010484; doi:10.1371/journal.pone.0096799)
Supplement: File S1 — Contains the following files: Figure S1. In vivo assays to select the best anti-TGF-β inhibitor. (A) A plasmid encoding IL-12 (pIL-12) was administered to C57BL/6 mice by hydrodynamic injection together with pApo, pSpP144, or pApoLinkerP144. Four days later, IFN-γ serum levels were quantified by ELISA. **p<0.01. (B) BALB/c mice were immunized with AH-1 peptide emulsified in incomplete Freund's adjuvant. At day 7 after immunization, mice received plasmids pApoA-I, pSpP144 or pApoLinkerP144 via hydrodynamic injection. Seven days later, mice were injected s.c. with 2.5×10^5^ CT26 tumor cells. Results are displayed as Kaplan-Meier plot of tumor occurrence. Treatment groups were compared using the log-rank test. Data are representative of one of two independent experiments (n = 6 mice/group). *p<0.05.**p<0.01. Figure S2. Inhibitory effect of ApoLinkerpP144 on TGF-β signaling. 5×10^5^ MC38 cells were plated in a 6-well plates. After an overnight incubation, cells were left untreated or treated with 100 pM TGF-β with or without 100 µg/ml rApoLinkerP144. 60 minutes later, cells were harvested and Western blot analysis was performed to determine the phosphorylation status of Smad2, Smad3 and Smad1/5/8. Figure S3. Scavenger receptor class B type I (SRB1) expression on MC38 tumor cell line. 3×10^5^ tumor cells were cultured in 6-well plates for 48 hours. Then, cells were harvested and stained to show SRB1 surface expression by flow cytometry analysis. Expression was compared with isotype control. Data are representative of two independent experiments. Figure S4. Antitumor efficacy of P144. 5×10^5^ MC38 colon carcinoma cells were intrasplenically injected. The same day, we started a daily treatment with vehicle (carbonate buffer pH 9.5) or 200 µg P144 in carbonate buffer (pH 9,5) for 15 days (n = 6/group). Mice were sacrificed at day 15 after tumor cell inoculation and tumor area in the liver was measured quantifying the pixels over a threshold color using Matlab software. Mean±SEM *, [file pone.0096799.s001.doc]

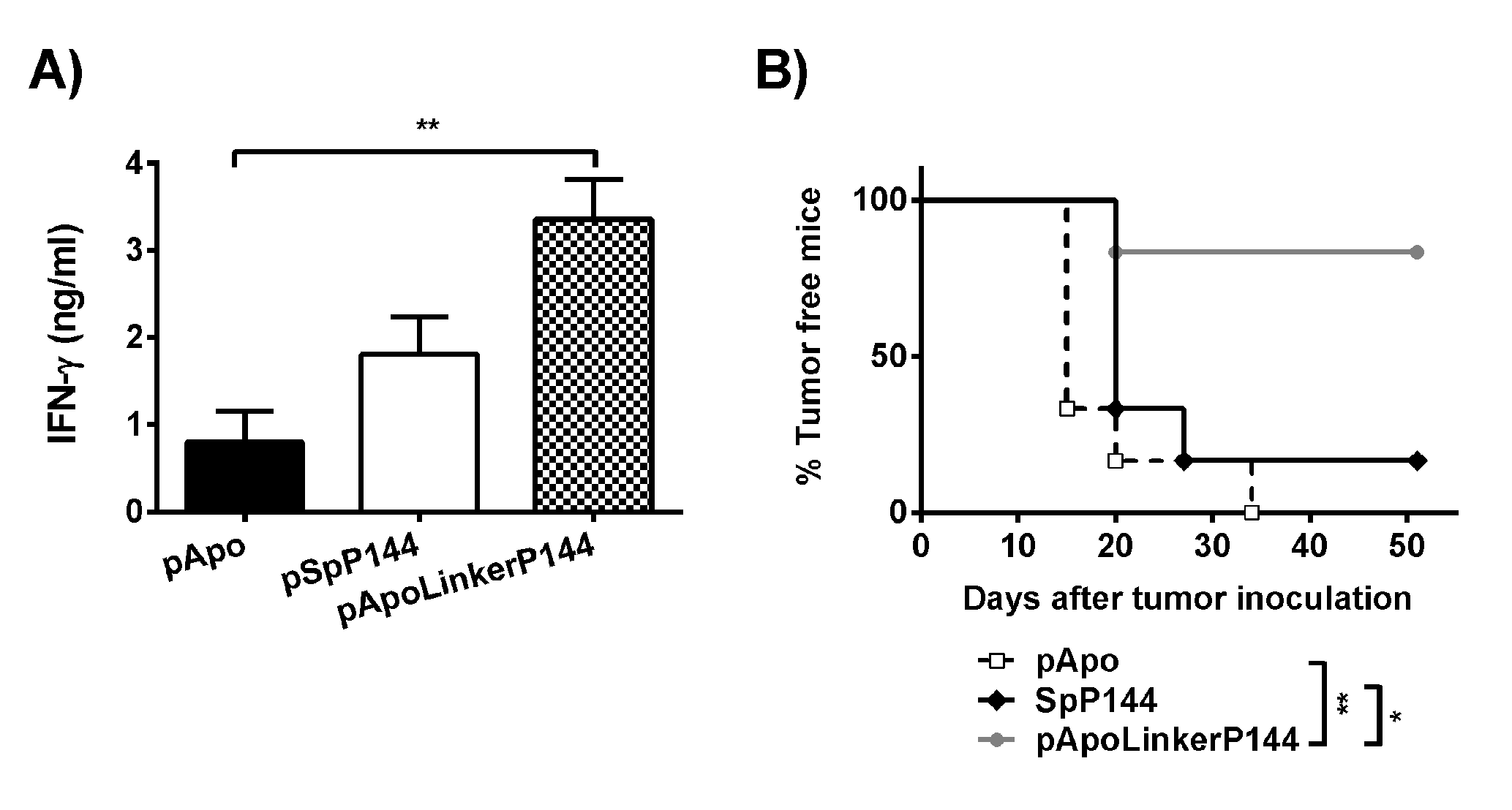


**Figure S1.**


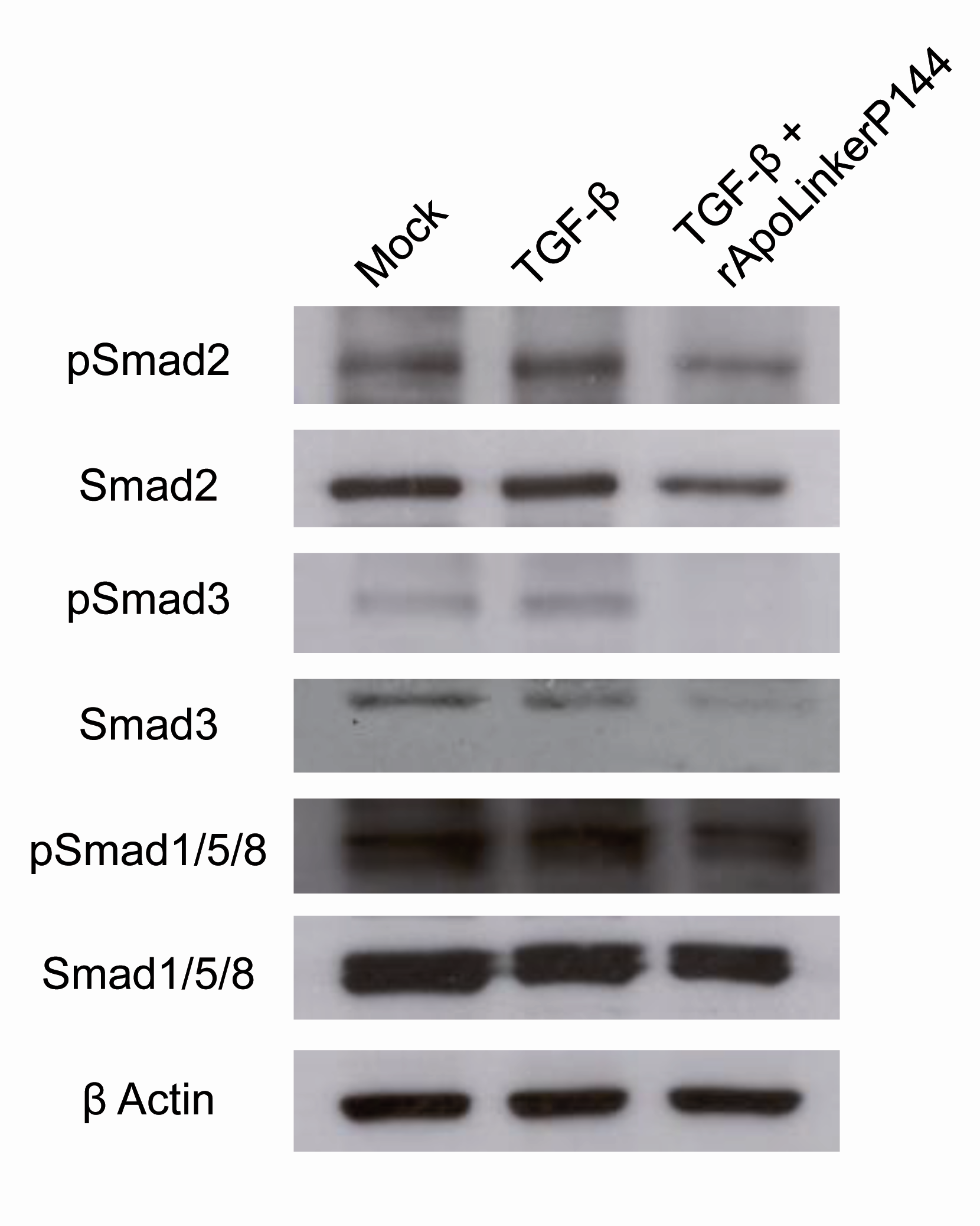


**Figure S2.**

MC38 isotype control Ab

MC38 SRB1 Ab

**Figure S3.**

**Figure S4.**


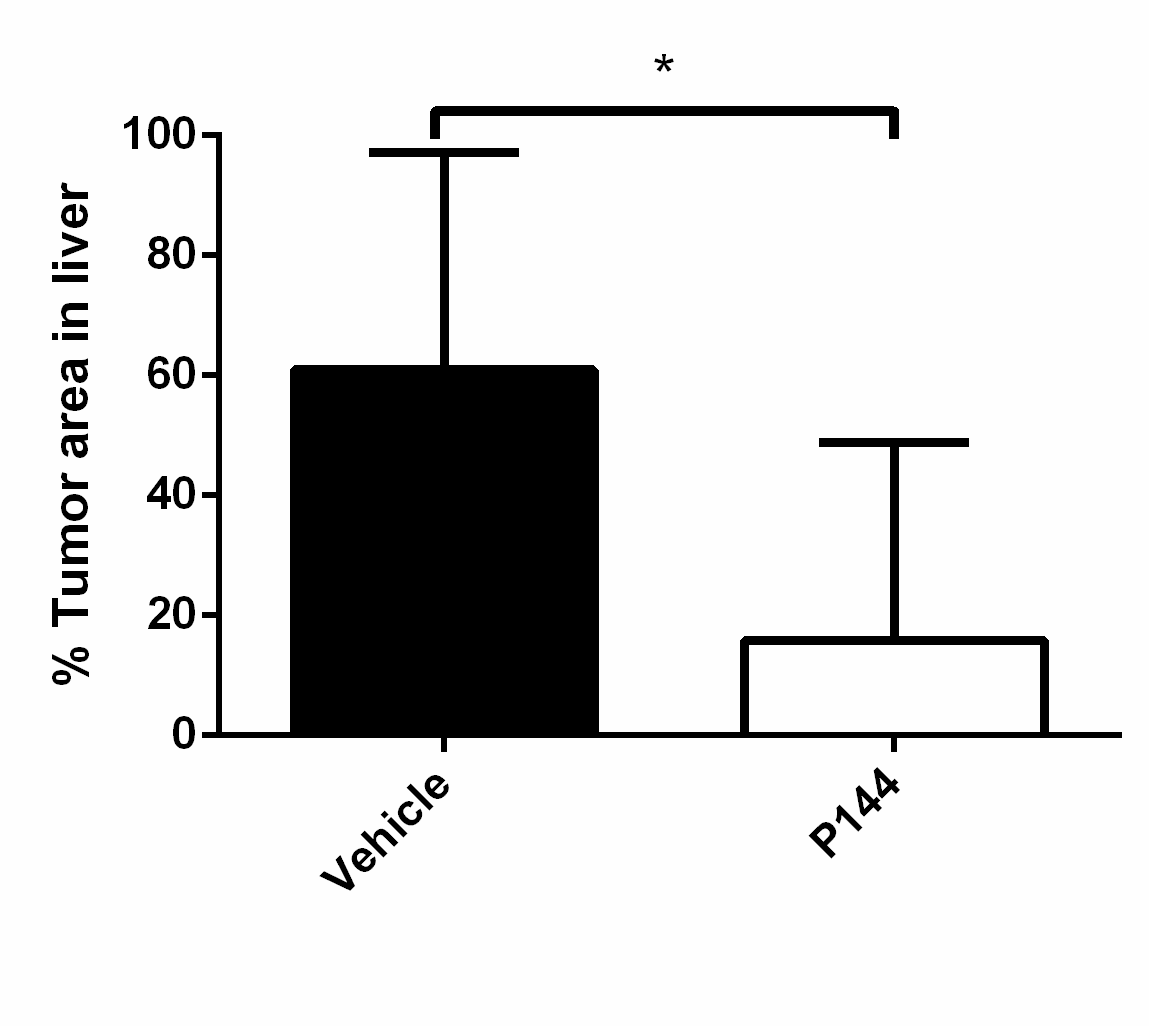


**Figure S5.**


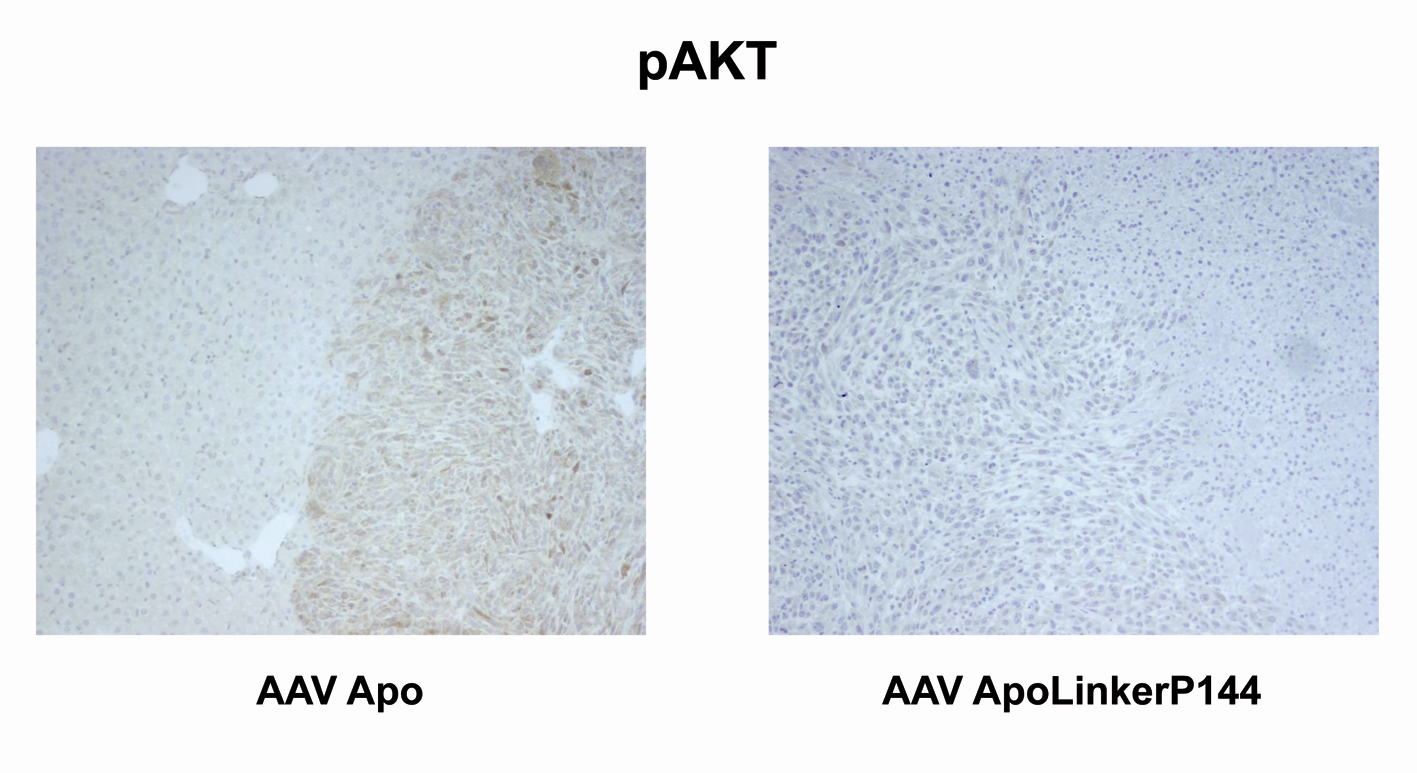


**Table S1.**

| ***Primer name*** | ***Sequence*** |
| --- | --- |
| *FwATGmApoAI* | 5’ ATGAAAGCTGTGGTGCTGGC 3’ |
| *RvSPmApoAIp144* | 5’TCATTCTGCATCATGGCCCAGATTATCGAGGCGTCCAGCGAGG  TTTGCTGCCAGAAATGCCG 3’ |
| *RvmApoAIp144* | 5'-TCAATTCTGCATCATGGCCCAGATTATCGAGGCGTCCAGCGAGG TGGGCGCGCCCTGGGC-3’ |
| *FwLINKERp144* | 5’CGCGCCGGCACCAGCAGAAACAAAAGCAGAACCAATGACAAC  CTCGCTGGACGCCTCGATAATCTGGGCCATGATGCAGAATTGAGC 3’ |
| *RvLINKERp144* | 5’ggccgctcaattctgcatcatggcccagattatcgaggcgtcc  agcgaggttgtcattggttctgcttttgtttctgctggtgccgg 3’ |
| *FGFr1 Fw* | 5´GCAGAGCATCAACTGGCTG 3´ |
| *FGFr1 Rv* | 5´ GGAGAAGTAGGTGGTATCGCTG 3´ |
| *Periostin Fw* | 5´ AATGCTGCCCTGGCTATATG 3´ |
| *Periostin Rv* | 5´ GCATGACCCTTTTCCTTCAA 3´ |
| *MMP9 Fw* | 5´ CGGTTGAAGCAAAGAAGGAG 3´ |
| *MMP9 Rv* | 5´ CCAAACCTTCAAAGGCCTCA 3´ |
| *MMP2 Fw* | 5´ TTTGCTCGGGCCTTAAAAGTAT 3´ |
| *MMP2 Rv* | 5´ CCATCAAACGGGTATCCATCTC 3´ |
| *COX-2 Fw* | 5´ GCCAATAGAACTTCCAATCCGTATAT 3´ |
| *COX-2 Rv* | 5´ TGGTCGGTTTGATGTTACTGTTG 3´ |
| *H3F3A-S444pan* | 5´AAAGCCGCTCGCAAGAGTGCG 3´ |
| *H3F3A-A665pan* | 5´ACTTGCCTCCTGCAAAGCAC 3´ |
| *IFNγ Fw* | 5´TCAAGTGGCATAGATGTGGAA 3´ |
| *IFNγ Rv* | 5´TGGCTCTGCAGGATTTTCATG 3´ |
| *GM-CSF Fw* | 5´ AAGGGTCCTGAGGAGGATGTG 3´ |
| *GM-CSF Rv* | 5´ GAGGTTCAGGGCTTCTTTGA 3´ |
| *FoxP3 Fw* | 5´GTTTCTCAAGCACTGCCAAG 3´ |
| *FoxP3 Rv* | 5´TGTGGAAGAACTCTGGGAAG 3´ |
